# Stoichiometric and polymorphic salt of imidazolium picrate monohydrate

**DOI:** 10.1107/S2056989017016401

**Published:** 2017-11-17

**Authors:** Ling-li Liu

**Affiliations:** a Renmin Hospital of Wuhan University, Wuhan 430060, People’s Republic of China

**Keywords:** crystal structure, co-crystal salt, imidazole, picric acid

## Abstract

The asymmetric unit of the title co-crystal salt, 1*H*-imidazol-3-ium 2,4,6-tri­nitro­phenolate monohydrate, C_4_H_7_N_2_^+^·C_6_H_2_N_3_O_7_^−^·H_2_O, contains one imidazolium cation, one picrate anion and one solvent water mol­ecule of crystallization. The phenolic proton has been transferred to an imidazole N atom. In the crystal, the components are linked by N—H⋯O and O—H⋯O hydrogen bonds into a three-dimensional network which is further consolidated by weak C—H⋯O hydrogen bonds. In addition, π–π stacking inter­actions occur between pairs of imidazolium cations and picrate anions. If only the classical N—H⋯O and O—H⋯O hydrogen bonds are considered, the component ions are linked into a three-dimensional threefold inter­penetrating network of the topological type **utp** [or (10,3)-*d*]. Hirshfeld surface analysis indicates the crystal structure is mainly stabilized by H⋯·O contacts of the hydrogen bonds.

## Abstract 2

An aqueous 1:1 co-crystal salt formed from imidazole and picric acid was obtained in methanol solution. A three-dimensional hydrogen-bonded network is formed in the crystal by N—H⋯O, O—H⋯O and C—H⋯O hydrogen bonds and is further consolidated by π–π stacking inter­actions between pairs of imidazolium cations and picrate anions.

## Chemical context   

**Figure 1:**
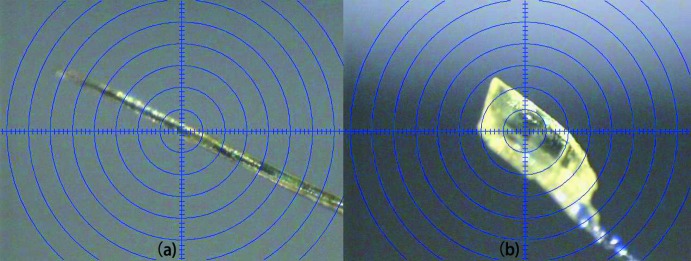
The morphologies of the two mol­ecular salts: needle (*a*) of (I) and block (*b*) of the crystal structure reported by Moreno-Fuquen *et al.* (2011 ▸).

Co-crystallization, which is the crystallization of more than one solid component into a new compound, is widely involved in the research fields of active pharmaceuticals (Aitipamula *et al.*, 2015 ▸; Weyna *et al.*, 2012 ▸; Robinson, 2010 ▸; Arenas-García *et al.*, 2010 ▸) and crystal engineering (Manoj *et al.*, 2014 ▸). Imidazole is an often used inter­mediate in pharmaceutical and chemical synthesis. Its crystallization characteristics can facilitate organic synthesis and theoretical prediction. Picric acid is a strong organic proton-donating reagent which can favor the crystallization of some basic organic complexes. By controlling one specific crystallization condition such as solvent, temperature, pressure or molar ratio of the raw materials, some polymorphs can be obtained with the same ingredients. For instance, two imidazolium picrate co-crystal salts have been reported that were crystallized from chloro­form (Soriano-García *et al.*, 1990 ▸) or dry aceto­nitrile (Moreno-Fuquen *et al.*, 2011 ▸). The crystal packing in these two analogs is completely different because of their different stoichiometric compositions. In order to further research the factors affecting this crystallization process, the crystallization solvent has been adjusted to be methanol (95%). Inter­estingly, some yellow needle-shaped crystals were obtained after two days on the side of the vessel and when the solvent had almost evaporated, several yellow block-shaped crystals formed at the bottom of the vessel (Fig. 1 ▸ *a* and 1*b*). The results of X-ray diffraction indicates that the structure of the block-shaped crystals is the same as that reported by Moreno-Fuquen *et al.* (2011 ▸). Herein, the crystal structure of the needle-shaped crystals is reported.
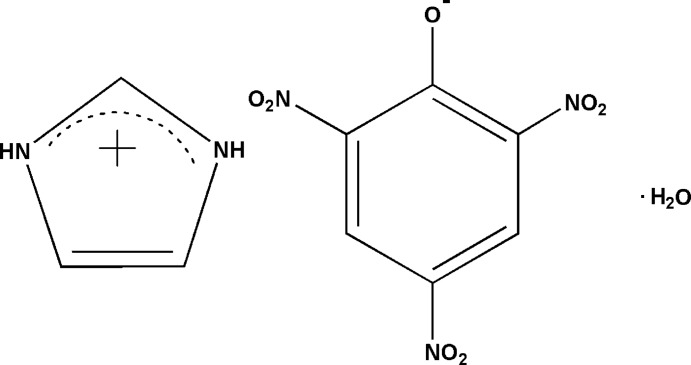


## Structural commentary   

**Figure 2:**
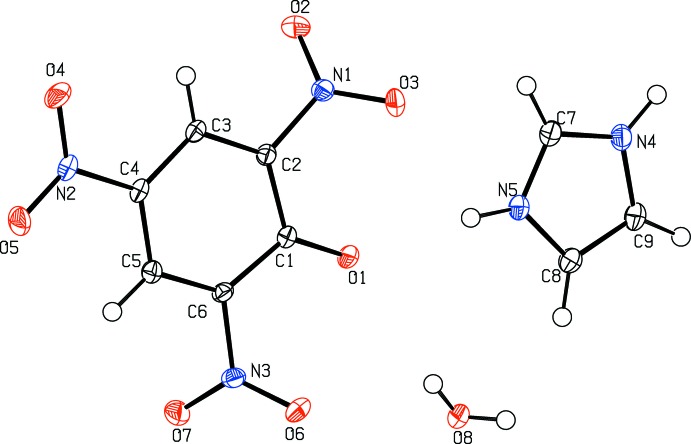
Mol­ecular structure of (I), showing the atom-numbering scheme. Displacement ellipsoids are drawn at the 50% probability level.

The asymmetric unit of the title compound (I) contains one imidazolium cation, a picrate anion and one solvent water mol­ecule of crystallization (Fig. 2 ▸). The phenolic proton has been transferred to a imidazole nitro­gen atom, forming the solvated 1:1 co-crystal salt. In the picrate anion of (I), the C—O_phenol_ bond distance is 1.250 (2) Å, which is shorter by *ca* 0.08 Å than the value of 1.33 (2) Å in the protonated species (Bertolasi *et al.*, 2011 ▸). Also, the two neighboring C—C bonds [1.447 (3) Å for C1—C2 and C1—C6], are significantly different from those in a benzene ring with delocalized C C bonds. The C2—C1—C6 angle [110.56 (16)°] is smaller by *ca* 11.3° than the averaged value of the other five ring inner angles [121.8 (1)°]. This deviation of bonds and angles can mainly be attributed to the electron-withdrawing effects of the three nitro groups, which can delocalize the negative charge on the phenolate O1 atom over the whole π-conjugated system. The nitro groups, N1/O2/O3, N2/O4/O5 and N3/O6/O7, are twisted from the central benzene ring by dihedral angles of 43.3 (3), 4.2 (3) and 48.5 (3)°, respectively. In the imidazolium cation, the C7—N4 [1.329 (3) Å] and C7—N5 [1.331 (3) Å] bond distances are the same due to the delocalizing effect and similar to those observed in other co-crystal salts (Soriano-García *et al.*, 1990 ▸; Moreno-Fuquen *et al.*, 2011 ▸).

## Supra­molecular features   

**Figure 3:**
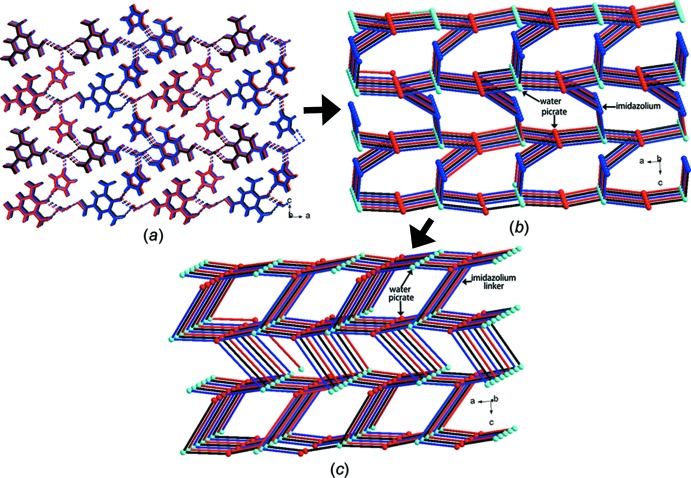
Part of the crystal structure of (I), showing (*a*) the formation of the three-dimensional hydrogen-bonded network by N—H⋯O and O—H⋯O hydrogen bonds as dashed lines, (*b*) the simplified network when picrate, water and imidazolium ions are considered as 3-, 3- and 2-connected nodes, respectively, and (*c*) the simplified **utp** network.

In the crystal of (I), the three components are linked into a three-dimensional network by N—H⋯O and O—H⋯O hydrogen bonds (Table 1 ▸, Fig. 3 ▸). In order to understand the structure simply, we can analyze it in the terms below. Firstly, the imidazolium cations, picrate anions and water mol­ecules are linked by each three N—H⋯O and three O—H⋯O hydrogen bonds, forming a three-dimensional framework structure (Fig. 3 ▸; Spek, 2003 ▸, 2009 ▸). It is worthy mentioning that if both the water mol­ecule and the picrate anion are regarded as 3-connected nodes by hydrogen-bonding and the imidazole cation as a 2-connected node, then the three-dimensional framework can be viewed topologically as a 3-connected **utp** network with a short Schläfli symbol of (10^3^)-*d* (Blatov *et al.*, 2014 ▸; Baburin & Blatov, 2007 ▸) (Fig. 3 ▸). Secondly, the three-dimensional hydrogen-bonded framework is consolidated by π–π inter­actions between pairs of imidazolium cations and picrate anions, both with centroid-to-centroid distances of 3.553 (4) Å, and weak inter­molecular C—H⋯O inter­actions (Table 1 ▸). It should be mentioned that the short O2⋯O7(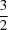 − *x*, *y* − 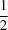, 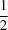 + *z*) contact of 2.837 (4) Å may be the result of an inclined NO_2_⋯π(NO_2_) inter­action (Daszkiewicz, 2013 ▸)

## Hirshfeld surface analysis   

**Figure 4:**
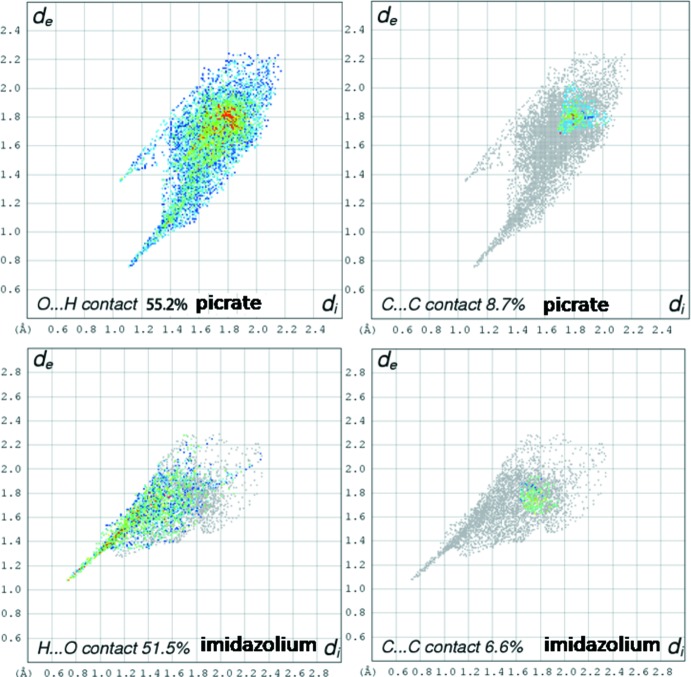
Fingerprint plots of co-crystal salt (I) showing the percentage of the O⋯H and C⋯C contacts around the environment of picrate and imidazolium ions.

An alternative way to asses the inter­molecular inter­actions qu­anti­tatively around one specific mol­ecule is through Hirshfeld surface analysis (Wolff *et al.*, 2012 ▸; McKinnon *et al.*, 2004 ▸). The Hirshfeld surface can define the environment of each crystallographically independent mol­ecule within a crystal. Fingerprint plots (Fig. 4 ▸) and show that for the picrate anion 55.2% and 8.8% of the area is concerned with the O⋯H (hydrogen-bonding) and C⋯C (π–π inter­action) contacts, respectively. In the imidazole cation, 51.5% and 9.7% of the area is concerned with the the O⋯H (hydrogen-bonding) and C⋯C/N (π–π inter­action) contacts, respectively. This qu­anti­tative analysis of the inter­molecular inter­actions again shows that the three-dimensional network is defined mainly by hydrogen bonds.

## Database survey   

A search of the Cambridge Structural Database (CSD version 5.37 plus one update, Groom *et al.*, 2016 ▸) indicates some analogs have been reported, *viz*. BEZGEU (Dhanabal *et al.*, 2013 ▸), QAKYOS (Dutkiewicz *et al.*, 2011 ▸), QAKGUG (Moreno-Fuquen *et al.*, 2011 ▸) and SEZREU (Soriano-García *et al.*, 1990 ▸). A structural comparison between these compounds indicates that the two nitro­gen atoms in the imidazolium cations are preferably hydrogen-bonded to the picrate anions, in which they can be in a bifurcated or a linear mode. For instance, in the 1:1 organic salt imidazolium picrate (QAKGUG; Moreno-Fuquen *et al.*, 2011 ▸), the imidazole N7 atom is linearly hydrogen-bonded to the phenolate oxygen atom O1. However, in the 1:2 salt (SEZREU; Soriano-García *et al.*, 1990 ▸), the imidazole N1 atom is involved in bifuracted hydrogen bonding to the phenolate O1 and nitro O2 atoms. Both of these compounds crystallize in orthorhombic space groups (*Pbca* or *P*2_1_2_1_2_1_). Further research about polymorphism in this system is being carried out in our lab.

## Synthesis and crystallization   

All the reagents and solvents were used as obtained without further purification. Equivalent molar amounts of imidazole (1.0 mmol, 68.0 mg) and picric acid (1.0 mmol, 229.0 mg) were dissolved in 95% methanol (40.0 ml). The mixture was stirred for half an hour at ambient temperature and then filtered. The resulting yellow solution was kept in air for two weeks. Yellow needle-shaped crystals of (I) suitable for single-crystal X-ray diffraction analysis formed on the side of the vessel after two days. The crystals were separated manually (yield: 75%, *ca* 0.24 g).

## Refinement   

Crystal data, data collection and structure refinement details of compound (I) are summarized in Table 2 ▸. H atoms bonded to C atoms were positioned geometrically with C—H = 0.93 Å (aromatic) and refined in riding mode [*U*_iso_(H) = 1.2*U*_eq_(C)]. H atoms bonded to N and O atoms were found in difference-Fourier maps and refined freely with constraints of *U*_iso_(H)= 1.2*U*_eq_(N) or 1.5*U*_eq_(N).

## Supplementary Material

Crystal structure: contains datablock(s) global, I. DOI: 10.1107/S2056989017016401/lh5861sup1.cif

Structure factors: contains datablock(s) I. DOI: 10.1107/S2056989017016401/lh5861Isup2.hkl

Supporting information file. DOI: 10.1107/S2056989017016401/lh5861Isup3.cml

CCDC reference: 1585713

Additional supporting information:  crystallographic information; 3D view; checkCIF report

## Figures and Tables

**Table 1:** Hydrogen-bond geometry (Å, °)

*D*—H⋯*A*	*D*—H	H⋯*A*	*D*⋯*A*	*D*—H⋯*A*
N4—H4⋯O8^i^	0.93 (3)	1.83 (3)	2.763 (3)	172 (3)
N5—H5*A*⋯O1^ii^	0.89 (3)	1.94 (3)	2.812 (2)	165 (3)
N5—H5*A*⋯O3^ii^	0.89 (3)	2.46 (3)	2.956 (3)	116 (2)
O8—H8*A*⋯O4^iii^	0.82 (4)	2.29 (4)	3.034 (2)	151 (3)
O8—H8*B*⋯O1^ii^	0.88 (4)	2.04 (4)	2.899 (2)	165 (3)
O8—H8*B*⋯O6^ii^	0.88 (4)	2.44 (4)	2.943 (3)	117 (3)
C5—H5⋯O2^iv^	0.95	2.49	3.383 (3)	157
C7—H7⋯O5^v^	0.95	2.37	3.187 (3)	144
C8—H8⋯O4^vi^	0.95	2.44	3.188 (3)	135
C8—H8⋯O8	0.95	2.53	3.255 (3)	133
C9—H9⋯O7^vii^	0.95	2.48	3.349 (3)	152

Symmetry codes: (i) 
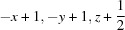
; (ii) 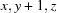; (iii) 
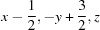
; (iv) 
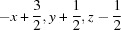
; (v) 
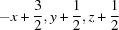
; (vi) 
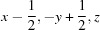
; (vii) 
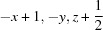
.

**Table 2:** Experimental details

Crystal data	
Chemical formula	C_3_H_5_N_2_^+^·C_6_H_2_N_3_O_7_^−^·H_2_O
*M*_r_	315.21
Crystal system, space group	Orthorhombic, *P* *n* *a*2_1_
Temperature (K)	100
*a*, *b*, *c* (Å)	21.577 (11), 3.5533 (18), 16.096 (8)
*V* (Å^3^)	1234.0 (11)
*Z*	4
Radiation type	Mo *K*α
μ (mm^−1^)	0.15
Crystal size (mm)	0.40 × 0.04 × 0.02

Data collection	
Diffractometer	Bruker APEXII CCD
Absorption correction	Multi-scan (*SADABS*; Bruker, 2001 ▸)
*T*_min_, *T*_max_	0.937, 0.997
No. of measured, independent and observed [*I* > 2σ(*I*)] reflections	11664, 3837, 3367
*R*_int_	0.038
(sin θ/λ)_max_ (Å^−1^)	0.724

Refinement	
*R*[*F* ^2^ > 2σ(*F* ^2^)], *wR*(*F* ^2^), *S*	0.035, 0.086, 1.04
No. of reflections	3837
No. of parameters	211
No. of restraints	1
H-atom treatment	H atoms treated by a mixture of independent and constrained refinement
Δρ_max_, Δρ_min_ (e Å^−3^)	0.35, −0.22

Computer programs: *APEX2* and *SAINT* (Bruker, 2001 ▸), *SHELXS97* and *SHELXTL* (Sheldrick, 2008 ▸), *SHELXL2014* (Sheldrick, 2015 ▸) and *DIAMOND* (Brandenburg, 2006 ▸).

## supplementary crystallographic information

### Crystal data

**Table:**

C_3_H_5_N_2_^+^·C_6_H_2_N_3_O_7_^−^·H_2_O	*D*_x_ = 1.697 Mg m^−^^3^
*M**_r_* = 315.21	Mo *K*α radiation, λ = 0.71073 Å
Orthorhombic, *P**n**a*2_1_	Cell parameters from 4015 reflections
*a* = 21.577 (11) Å	θ = 2.3–31.2°
*b* = 3.5533 (18) Å	µ = 0.15 mm^−^^1^
*c* = 16.096 (8) Å	*T* = 100 K
*V* = 1234.0 (11) Å^3^	Needle, yellow
*Z* = 4	0.40 × 0.04 × 0.02 mm
*F*(000) = 648	

### Data collection

**Table:**

Bruker APEXII CCD diffractometer	3367 reflections with *I* > 2σ(*I*)
φ and ω scans	*R*_int_ = 0.038
Absorption correction: multi-scan (SADABS; Bruker, 2001)	θ_max_ = 31.0°, θ_min_ = 1.9°
*T*_min_ = 0.937, *T*_max_ = 0.997	*h* = −28→30
11664 measured reflections	*k* = −4→5
3837 independent reflections	*l* = −23→22

### Refinement

**Table:**

Refinement on *F*^2^	Hydrogen site location: mixed
Least-squares matrix: full	H atoms treated by a mixture of independent and constrained refinement
*R*[*F*^2^ > 2σ(*F*^2^)] = 0.035	*w* = 1/[σ^2^(*F*_o_^2^) + (0.0494*P*)^2^ + 0.0144*P*] where *P* = (*F*_o_^2^ + 2*F*_c_^2^)/3
*wR*(*F*^2^) = 0.086	(Δ/σ)_max_ < 0.001
*S* = 1.04	Δρ_max_ = 0.35 e Å^−^^3^
3837 reflections	Δρ_min_ = −0.22 e Å^−^^3^
211 parameters	Absolute structure: Flack *x* determined using 1456 quotients [(*I*^+^)-(*I*^-^)]/[(*I*^+^)+(*I*^-^)] (Parsons *et al.*, 2013)
1 restraint	Absolute structure parameter: 0.4 (5)

### Special details

**Table:**

Geometry. All esds (except the esd in the dihedral angle between two l.s. planes) are estimated using the full covariance matrix. The cell esds are taken into account individually in the estimation of esds in distances, angles and torsion angles; correlations between esds in cell parameters are only used when they are defined by crystal symmetry. An approximate (isotropic) treatment of cell esds is used for estimating esds involving l.s. planes.

### Fractional atomic coordinates and isotropic or equivalent isotropic displacement parameters (Å^2^)

**Table:**

	*x*	*y*	*z*	*U*_iso_*/*U*_eq_	
C1	0.67480 (9)	−0.0182 (6)	0.64727 (12)	0.0122 (3)	
C2	0.72241 (9)	0.0454 (6)	0.70899 (11)	0.0125 (4)	
C3	0.78154 (9)	0.1698 (6)	0.69303 (11)	0.0131 (4)	
H3	0.8106	0.2010	0.7368	0.016*	
C4	0.79782 (9)	0.2488 (6)	0.61128 (12)	0.0128 (3)	
C5	0.75625 (8)	0.1917 (6)	0.54609 (12)	0.0133 (4)	
H5	0.7681	0.2377	0.4902	0.016*	
C6	0.69788 (9)	0.0674 (6)	0.56500 (12)	0.0125 (3)	
C8	0.48877 (9)	0.4718 (7)	0.77357 (13)	0.0172 (4)	
H8	0.4715	0.4535	0.7194	0.021*	
C9	0.46050 (9)	0.3770 (7)	0.84591 (13)	0.0181 (4)	
H9	0.4197	0.2809	0.8522	0.022*	
N1	0.70790 (8)	−0.0342 (5)	0.79547 (11)	0.0143 (3)	
N2	0.85824 (8)	0.4007 (5)	0.59405 (11)	0.0150 (3)	
N3	0.65576 (8)	0.0095 (5)	0.49511 (11)	0.0149 (3)	
C7	0.55452 (10)	0.5801 (7)	0.87530 (13)	0.0172 (4)	
H7	0.5908	0.6495	0.9049	0.021*	
N4	0.50257 (9)	0.4472 (6)	0.90866 (12)	0.0170 (3)	
H4	0.4957 (14)	0.398 (9)	0.9649 (19)	0.020*	
N5	0.54696 (8)	0.5991 (5)	0.79335 (11)	0.0157 (3)	
H5A	0.5763 (13)	0.682 (8)	0.7586 (18)	0.019*	
O1	0.62270 (6)	−0.1576 (5)	0.66176 (9)	0.0156 (3)	
O4	0.89440 (7)	0.4486 (5)	0.65267 (10)	0.0226 (3)	
O5	0.87159 (7)	0.4829 (5)	0.52189 (10)	0.0226 (4)	
O8	0.50725 (7)	0.6717 (6)	0.57776 (10)	0.0214 (3)	
H8A	0.4849 (16)	0.841 (10)	0.594 (2)	0.032*	
H8B	0.5441 (17)	0.745 (10)	0.595 (2)	0.032*	
O2	0.74665 (8)	−0.1992 (5)	0.83731 (10)	0.0227 (4)	
O3	0.65759 (7)	0.0718 (5)	0.82223 (10)	0.0219 (3)	
O6	0.60348 (7)	0.1417 (5)	0.50023 (10)	0.0222 (4)	
O7	0.67538 (7)	−0.1656 (5)	0.43500 (9)	0.0211 (3)	

### Atomic displacement parameters (Å^2^)

**Table:**

	*U*^11^	*U*^22^	*U*^33^	*U*^12^	*U*^13^	*U*^23^
C1	0.0097 (7)	0.0125 (9)	0.0143 (8)	0.0002 (6)	0.0006 (6)	0.0003 (7)
C2	0.0109 (8)	0.0138 (10)	0.0127 (8)	0.0000 (7)	0.0006 (6)	0.0008 (7)
C3	0.0099 (8)	0.0138 (10)	0.0157 (8)	0.0014 (7)	−0.0005 (6)	−0.0006 (7)
C4	0.0085 (7)	0.0128 (9)	0.0171 (8)	−0.0001 (6)	0.0008 (6)	−0.0002 (6)
C5	0.0115 (8)	0.0135 (10)	0.0148 (8)	−0.0003 (7)	0.0010 (6)	0.0006 (7)
C6	0.0101 (8)	0.0142 (10)	0.0134 (8)	−0.0012 (7)	−0.0020 (6)	0.0002 (7)
C8	0.0135 (9)	0.0198 (11)	0.0182 (9)	−0.0029 (7)	0.0000 (7)	−0.0001 (8)
C9	0.0125 (8)	0.0203 (11)	0.0215 (9)	−0.0020 (8)	0.0028 (7)	0.0004 (8)
N1	0.0133 (8)	0.0159 (9)	0.0138 (7)	−0.0034 (6)	−0.0006 (6)	−0.0001 (6)
N2	0.0097 (7)	0.0151 (9)	0.0203 (8)	0.0000 (6)	0.0013 (6)	0.0000 (7)
N3	0.0129 (8)	0.0181 (9)	0.0136 (7)	−0.0029 (6)	−0.0025 (5)	0.0031 (6)
C7	0.0132 (9)	0.0198 (11)	0.0185 (9)	0.0008 (7)	0.0013 (7)	−0.0004 (8)
N4	0.0143 (7)	0.0193 (9)	0.0176 (8)	0.0004 (7)	0.0034 (6)	0.0004 (7)
N5	0.0122 (8)	0.0184 (9)	0.0163 (7)	−0.0012 (6)	0.0021 (6)	0.0006 (7)
O1	0.0095 (6)	0.0210 (8)	0.0163 (6)	−0.0035 (5)	−0.0002 (5)	0.0011 (6)
O4	0.0112 (6)	0.0316 (10)	0.0248 (7)	−0.0045 (6)	−0.0036 (6)	0.0025 (7)
O5	0.0166 (7)	0.0318 (10)	0.0195 (7)	−0.0063 (6)	0.0057 (6)	−0.0003 (6)
O8	0.0122 (7)	0.0340 (10)	0.0181 (7)	0.0016 (7)	−0.0005 (5)	−0.0032 (7)
O2	0.0228 (8)	0.0294 (10)	0.0158 (7)	0.0039 (7)	−0.0032 (6)	0.0040 (7)
O3	0.0141 (7)	0.0333 (10)	0.0182 (7)	−0.0005 (6)	0.0056 (6)	−0.0026 (6)
O6	0.0124 (7)	0.0318 (10)	0.0224 (7)	0.0042 (6)	−0.0030 (5)	0.0031 (7)
O7	0.0187 (7)	0.0287 (10)	0.0159 (6)	−0.0031 (6)	−0.0002 (6)	−0.0056 (6)

### Geometric parameters (Å, º)

**Table:**

C1—O1	1.250 (2)	C9—N4	1.381 (3)
C1—C2	1.447 (3)	C9—H9	0.9500
C1—C6	1.447 (3)	N1—O2	1.223 (2)
C2—C3	1.374 (3)	N1—O3	1.227 (2)
C2—N1	1.455 (3)	N2—O5	1.232 (2)
C3—C4	1.391 (3)	N2—O4	1.236 (2)
C3—H3	0.9500	N3—O6	1.225 (2)
C4—C5	1.395 (3)	N3—O7	1.226 (3)
C4—N2	1.438 (3)	C7—N4	1.329 (3)
C5—C6	1.369 (3)	C7—N5	1.331 (3)
C5—H5	0.9500	C7—H7	0.9500
C6—N3	1.461 (3)	N4—H4	0.93 (3)
C8—C9	1.357 (3)	N5—H5A	0.89 (3)
C8—N5	1.372 (3)	O8—H8A	0.82 (4)
C8—H8	0.9500	O8—H8B	0.88 (4)
			
O1—C1—C2	124.91 (18)	C8—C9—H9	126.7
O1—C1—C6	124.28 (17)	N4—C9—H9	126.7
C2—C1—C6	110.56 (16)	O2—N1—O3	123.96 (18)
C3—C2—C1	125.53 (17)	O2—N1—C2	118.23 (17)
C3—C2—N1	116.18 (16)	O3—N1—C2	117.81 (17)
C1—C2—N1	118.28 (17)	O5—N2—O4	122.65 (17)
C2—C3—C4	118.43 (17)	O5—N2—C4	118.87 (17)
C2—C3—H3	120.8	O4—N2—C4	118.47 (16)
C4—C3—H3	120.8	O6—N3—O7	124.46 (18)
C3—C4—C5	121.33 (17)	O6—N3—C6	117.85 (18)
C3—C4—N2	119.15 (17)	O7—N3—C6	117.69 (17)
C5—C4—N2	119.50 (17)	N4—C7—N5	108.37 (19)
C6—C5—C4	118.12 (17)	N4—C7—H7	125.8
C6—C5—H5	120.9	N5—C7—H7	125.8
C4—C5—H5	120.9	C7—N4—C9	108.85 (19)
C5—C6—C1	126.00 (17)	C7—N4—H4	126.4 (19)
C5—C6—N3	116.53 (17)	C9—N4—H4	124.7 (19)
C1—C6—N3	117.46 (16)	C7—N5—C8	108.99 (18)
C9—C8—N5	107.10 (19)	C7—N5—H5A	123.3 (18)
C9—C8—H8	126.4	C8—N5—H5A	127.7 (18)
N5—C8—H8	126.4	H8A—O8—H8B	102 (3)
C8—C9—N4	106.69 (18)		
			
O1—C1—C2—C3	174.4 (2)	C3—C2—N1—O2	−42.2 (3)
C6—C1—C2—C3	0.0 (3)	C1—C2—N1—O2	136.7 (2)
O1—C1—C2—N1	−4.3 (3)	C3—C2—N1—O3	137.2 (2)
C6—C1—C2—N1	−178.67 (19)	C1—C2—N1—O3	−44.0 (3)
C1—C2—C3—C4	1.1 (3)	C3—C4—N2—O5	−177.4 (2)
N1—C2—C3—C4	179.82 (18)	C5—C4—N2—O5	1.2 (3)
C2—C3—C4—C5	−2.3 (3)	C3—C4—N2—O4	1.5 (3)
C2—C3—C4—N2	176.28 (19)	C5—C4—N2—O4	−179.89 (19)
C3—C4—C5—C6	2.3 (3)	C5—C6—N3—O6	−132.1 (2)
N2—C4—C5—C6	−176.28 (19)	C1—C6—N3—O6	49.1 (3)
C4—C5—C6—C1	−1.1 (3)	C5—C6—N3—O7	47.3 (3)
C4—C5—C6—N3	−179.77 (19)	C1—C6—N3—O7	−131.5 (2)
O1—C1—C6—C5	−174.5 (2)	N5—C7—N4—C9	0.3 (3)
C2—C1—C6—C5	0.0 (3)	C8—C9—N4—C7	0.0 (3)
O1—C1—C6—N3	4.2 (3)	N4—C7—N5—C8	−0.6 (3)
C2—C1—C6—N3	178.65 (18)	C9—C8—N5—C7	0.6 (3)
N5—C8—C9—N4	−0.3 (3)		

### Hydrogen-bond geometry (Å, º)

**Table:**

*D*—H···*A*	*D*—H	H···*A*	*D*···*A*	*D*—H···*A*
N4—H4···O8^i^	0.93 (3)	1.83 (3)	2.763 (3)	172 (3)
N5—H5*A*···O1^ii^	0.89 (3)	1.94 (3)	2.812 (2)	165 (3)
N5—H5*A*···O3^ii^	0.89 (3)	2.46 (3)	2.956 (3)	116 (2)
O8—H8*A*···O4^iii^	0.82 (4)	2.29 (4)	3.034 (2)	151 (3)
O8—H8*B*···O1^ii^	0.88 (4)	2.04 (4)	2.899 (2)	165 (3)
O8—H8*B*···O6^ii^	0.88 (4)	2.44 (4)	2.943 (3)	117 (3)
C5—H5···O2^iv^	0.95	2.49	3.383 (3)	157
C7—H7···O5^v^	0.95	2.37	3.187 (3)	144
C7—H7···O3^ii^	0.95	2.47	2.955 (3)	112
C8—H8···O4^vi^	0.95	2.44	3.188 (3)	135
C8—H8···O8	0.95	2.53	3.255 (3)	133
C9—H9···O7^vii^	0.95	2.48	3.349 (3)	152

Symmetry codes: (i) −*x*+1, −*y*+1, *z*+1/2; (ii) *x*, *y*+1, *z*; (iii) *x*−1/2, −*y*+3/2, *z*; (iv) −*x*+3/2, *y*+1/2, *z*−1/2; (v) −*x*+3/2, *y*+1/2, *z*+1/2; (vi) *x*−1/2, −*y*+1/2, *z*; (vii) −*x*+1, −*y*, *z*+1/2.
